# Atypical lipoma of the right piriformis muscle: a case report and review of the literature

**DOI:** 10.1186/s13256-024-04507-1

**Published:** 2024-03-31

**Authors:** Xiao Qiu, Xiaoyong Luo, Renmei Wu

**Affiliations:** Department of Ultrasound, Suining Central Hospital, 127 Desheng West Road, Suining, Sichuan China

**Keywords:** Lipoma, Piriformis muscle, Sciatica

## Abstract

**Background:**

Piriformis muscle mass is rare, which is particular for intrapiriformis lipoma. Thus far, only 11 cases of piriformis muscle mass have been reported in the English literature. Herein, we encountered one patient with intrapiriformis lipoma who was initially misdiagnosed.

**Case presentation:**

The patient is a 50-year-old Chinese man. He complained of osphyalgia, right buttock pain, and radiating pain from the right buttock to the back of the right leg. Both ultrasound and magnetic resonance imaging demonstrated a cyst-like mass in the right piriformis muscle. Ultrasonography-guided aspiration was performed on this patient first, but failed. He was then recommended to undergo mass resection and neurolysis of sciatic nerve. Surprisingly, final histology revealed the diagnosis of intrapiriformis lipoma. The patient exhibited significant relief of symptoms 3 days post-surgery.

**Conclusion:**

Diagnosis and differential diagnosis of radicular pain are potentially challenging but necessary. Atypical lipoma is prone to be misdiagnosed, especially in rare sites. It is notable for clinicians to be aware of the presence of intrapiriformis lipoma to avoid misdiagnosis and inappropriate treatment.

## Background

Piriformis syndrome (PS), also known as sciatic nerve outlet syndrome, caused by compression of the sciatic nerve by the piriformis muscle, is characterized by occasional sciatic-type pain, tingling, and numbness in the buttock along the sciatic nerve pathway down to the lower thigh and the calf [1]. The causes of PS are diversified, including inflammatory, traumatic, tumoral, and malformative factors [2, 3]. PS triggered by space-occupying lesions of the piriformis muscle is very rare. Up to date, only 11 cases of piriformis muscle mass have been reported in the English literature [4–10].

Lipomas are one of the most common mesenchymal neoplasms and can occur in any region of the body that contains fat component, including the subcutaneous soft tissues, mediastinum, retroperitoneum, bones, or along the gastrointestinal tract [11]. Intrapiriformis lipoma is rare and the diagnosis might be intractable when presenting atypical. In addition, misdiagnosis can lead to inappropriate treatment, which causes unsatisfactory outcomes. Here, we present a case of a large intrapiriformis lipoma that was initially misdiagnosed, highlighting that clinicians should be aware that intrapiriformis lipoma might harbor atypical manifestations upon examination.

## Case presentation

A 50-year-old Chinese man presented to the orthopedics department with chief complaints of osphyalgia, right buttock pain, and radiating pain from the right buttock to the back of the right leg. The right buttock pain was the most prominent symptom. The pain was accelerated by movement and relieved by lying supine, which induced abnormal walk in the patient. No previous relevant treatment or surgery was reported. Additionally, there was no significant relevant family or social history.

Lasegue’s sign and its strengthening test were positive. Physical examination also demonstrated the positive findings of right femoral nerve traction test and Faber test, and the limitation of right hip abduction was observed. Neurological examination of the lower limb did not demonstrate any loss of sensation or reduced muscle power in any of the nerve root distributions. Non-remarkable finding was revealed after the abdominal examination.

**Fig. 1:**
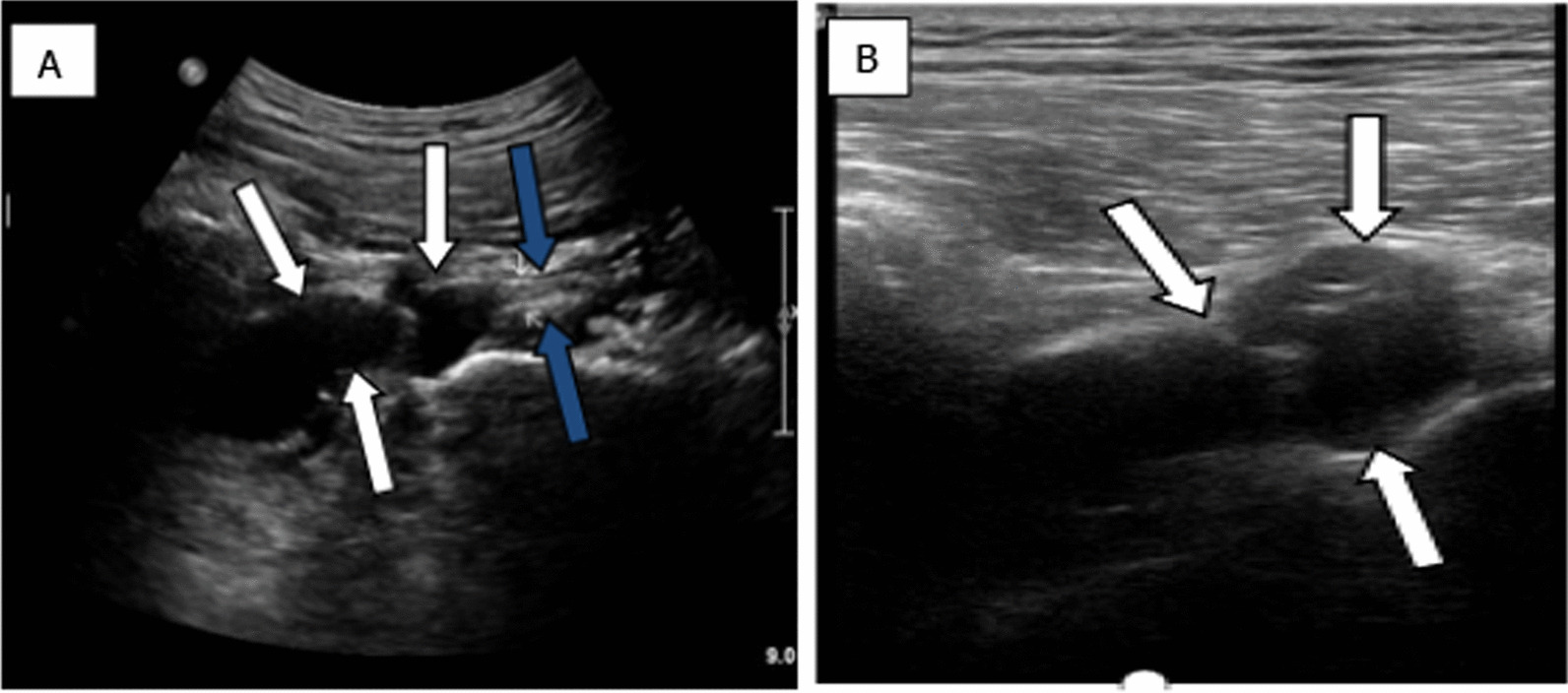
Sonographic examination showing a separate anechoic mass (white arrows) above the outlet of the right piriformis muscle pushing the right sciatic nerve (blue arrows). **A** Sagittal view(low- frequency probe); **B** Transverse view (high-frequency probe)

**Fig. 2:**
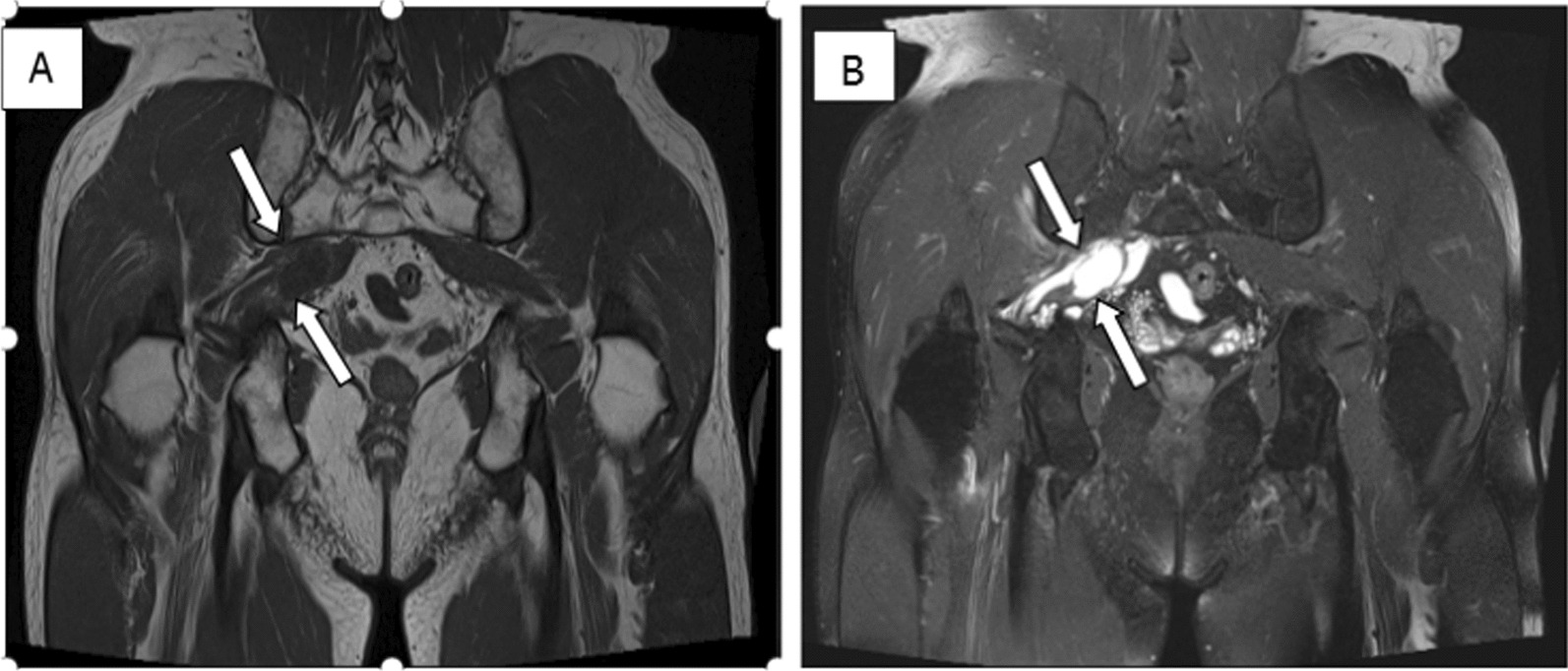
MRI demonstrating multiple cysts (arrows) in piriformis muscle region with long T1 (**A**) and long T2 (**B**) signals

As no apparent abnormalities were indicated upon the plain radiograph imaging of his lumbar spine, magnetic resonance imaging (MRI) scan of the lumbar/sacral area of the spine was then suggested, showing lumbar disc herniation (LDH), which did not account for the patient’s predominant right buttock pain. Thus, the musculoskeletal ultrasound (MSK-US) for the sciatic nerve scanning was performed, implying that the right sciatic nerve was pushed by an anechoic mass within the right piriformis muscle measuring 6 cm mediolateral, 2.3 cm anteroposterior, and 2.6 cm craniocaudal. The lobulated mass was cystic-like with regular margins and no posterior wall enhancement (Fig. 1). Subsequently, further MRI of the pelvis and ipsilateral hip indicated a cystic-like lesion in piriformis muscle region with low T1 signal and high T2 signal, and the maximum measurement was about 3.1 cm mediolateral and 2.2 cm anteroposterior (Fig. 2). Considering these results, a piriformis ganglion was suspected, and the differential diagnoses included hematoma, metastatic tumor, and so forth.

**Fig. 3:**
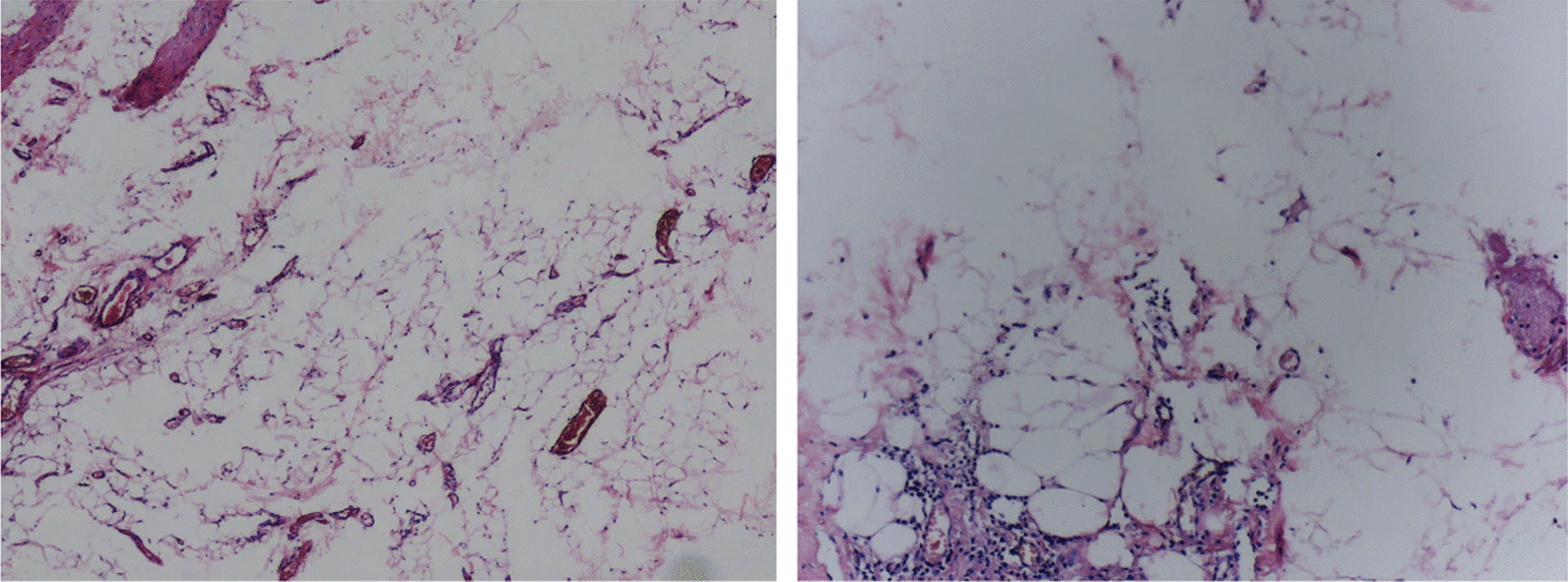
Under microscope inspection, removed specimen revealed fibrous adipose tissue within the lesion, consistent with lipoma

Aiming to achieve the final diagnosis, the ultrasonography-guided aspiration was conducted, but failed due to unextracted cystic fluid. In addition, significant resistance was encountered when injecting with physiological saline. As for the undefined nature of the mass and the associated serious symptoms, malignancy could not be excluded; the patient was suggested to undergo piriformis muscle mass resection and neurolysis of sciatic nerve. Operative finding showed the compression of right sciatic nerve by a fat-like mass at the lower margin of piriformis muscle measuring 5 cm mediolateral, 2 cm anteroposterior, and 2 cm craniocaudal. Final histology revealed that the lesion was fibrous adipose tissue, which was consistent with diagnosis of lipoma (Fig. 3). The patient exhibited significant relief of symptoms 3 days post-surgery. No recurrence of relevant symptoms was observed during 24-month follow-up period.

## Discussion

Lower back pain can present with radicular pain caused by lumbosacral nerve root pathology. As a major cause of lower back pain, sciatica, and radicular leg pain, LDH is usually the first considered diagnosis. Similarly, in our case LDH was initially considered according to the MRI of the lumbar/sacral spine. However, the primary pain in the right buttock of this patient was unexplained on the diagnosis of LDH.

PS, also known as sciatic nerve outlet syndrome, is a type of sciatic neuralgia caused by compression of the piriformis muscle on the sciatic nerve. Typical manifestations include buttock pain and radiating pain in the innervated area of the sciatic nerve. In general, etiologies are composed of traumatic bleeding, adhesions, scars, anatomical variations, and so forth [2]. Of note, intrapiriformis lesion enlarging the muscle may be the common cause of sciatic nerve compression-induced secondary PS, whereas PS triggered by space-occupying lesions of the piriformis muscle is very rare. To the best of our knowledge, only 11 cases have been reported in the literature thus far; these patients and our present case are summarized in Table 1 [4–10], among which only 1 case of intramuscular lipomas occurring within the piriformis muscle leading to secondary PS have been previously reported in the literature [6].

**Table 1:** Summary of piriformis mass in the literature

Authors	Year	Age/sex	Laterality	Size	Procedure	Diagnosis
O. Salar *et al*. [4]	2012	67/F	L	2.1 × 1.8 × 2.8 cm	Biopsy	Metastatic adenocarcinoma
Domínguez-Páez Miguel *et al* [5]	2012	29/F	R	–	Excision	Endometriosis
E. Drampalos *et al*. [6]	2014	48/F	L	7 × 3 × 6 cm	Excision	Intramuscular lipoma
J.H. Park *et al*. [7]	2016	32/F	R	3.4 × 2.0 cm	Ultrasonography-guided aspiration	Ganglionic cyst
J.H. Park *et al*. [7]	2016	39/M	L	5 × 1.6 cm	Ultrasonography-guided aspiration	Ganglionic cyst
Jan Lodin *et al*. [8]	2021	55/M	L	–	Hematoma evacuation	Hematoma
Naoko Sanuki *et al*. [9]	2022	74/F	L	–	Radiation therapy	Endometrial cancer
Naoko Sanuki *et al*. [9]	2022	44/F	L	–	Radiation therapy	Cervical cancer
Naoko Sanuki *et al*. [9]	2022	71/M	L	–	Radiation therapy	Rectal cancer
Naoko Sanuki *et al*. [9]	2022	80/M	L	–	Radiation therapy	Bladder cancer
Thomas Robert William Ward *et al* [10]	2022	36/F	L	–	Surgical exploration	Hematoma
Present case		50/M	R	6 × 2.3 × 2.6 cm	Excision	Intramuscular lipoma

Lipomas can be classified into superficial and deep lesions according to the location. Deep-seated lipomas are less common than superficial lipomas, which may be located under the muscle (submuscular), within the muscle (intramuscular), between the muscles (intermuscular), or on top of the muscle (supramuscular) [11]. Clinically, lipomas often present as asymptomatic slow-growing mass or swelling with no palpable mass. The application of ultrasound (US) in the examination of lipomas is very frequent. Usually, superficial lipomas might manifest as a hyperechoic solid mass without posterior acoustic enhancement or show as a isoechoic mass on gray-scale US. Compared with superficial lipomas, the deep-seated type can present as various US characteristics. In addition, few reports in the literature show the hypoechoic, isoechoic, or anechoic properties of deep ones [12–14]. However, the intrapiriformis lipoma in our case was featured as an anechoic lesion, usually indicated as cystic lesions. The MRI of the pelvis and ipsilateral hip showed the same signal characteristics as those of water on all sequences. Therefore, the lesion within piriformis muscle region was then misdiagnosed as ganglion and distinguished from neuschwannoma, liposarcoma, hematoma, lymphoma, metastatic tumor, and so on. Therefore, ultrasonography-guided aspiration was performed while noncystic fluid was extracted.

The echogenicity of lipomas may range from hyperechoic to anechoic, depending on the component percentage of connective tissue and other reflective interfaces presented within a lipoma [15]. It has been postulated that US and MRI appearance of lipomas are largely dependent on the internal cellularity, specifically on the proportion of fat and water within the lesion [16]. When the proportion of water in the lipoma is high, it may present the same imaging characteristics as this case.

Generally, intrapiriformis lipoma does not require treatment in the absence of symptoms, while for our case, considering the serious symptoms of this patient and undefined nature, even including malignancy, after series of examinations, surgical treatment was recommended. Fortunately, the patient showed significant relief of symptoms 3 days after surgery. No recurrence of associated symptoms was observed during 24-month follow-up period.

## Conclusion

Despite the potentially significant challenges for the diagnosis and differential diagnosis of radicular pain, it is highly necessary and essential. It is notable that medical practitioners should be aware of this condition and exclude space-occupying lesions of piriformis muscles when encountering patients presenting with radicular pain. Our case highlighted the atypical manifestations of lipomas in rare areas such as piriformis muscles, for which condition-adequate examinations should be performed and surgery might be finally suggested to reach the final diagnosis, thus avoiding misdiagnosis and inappropriate treatment and increasing the life quality of these patients.

## Data Availability

The authors of this manuscript are willing to provide additional information regarding the case report.

## Abbreviations

MSK-US: Musculoskeletal ultrasound
US: Ultrasound
MRI: Magnetic resonance imaging
PS: Piriformis syndrome
LDH: Lumbar disc herniation
